# Supplementary figures and images for: The Construction of a Multi-Gene Risk Model for Colon Cancer Prognosis and Drug Treatments Prediction
Source: Int J Mol Sci. 2024 Apr 2;25(7):3954. doi: 10.3390/ijms25073954 (PMC11011764; doi:10.3390/ijms25073954)

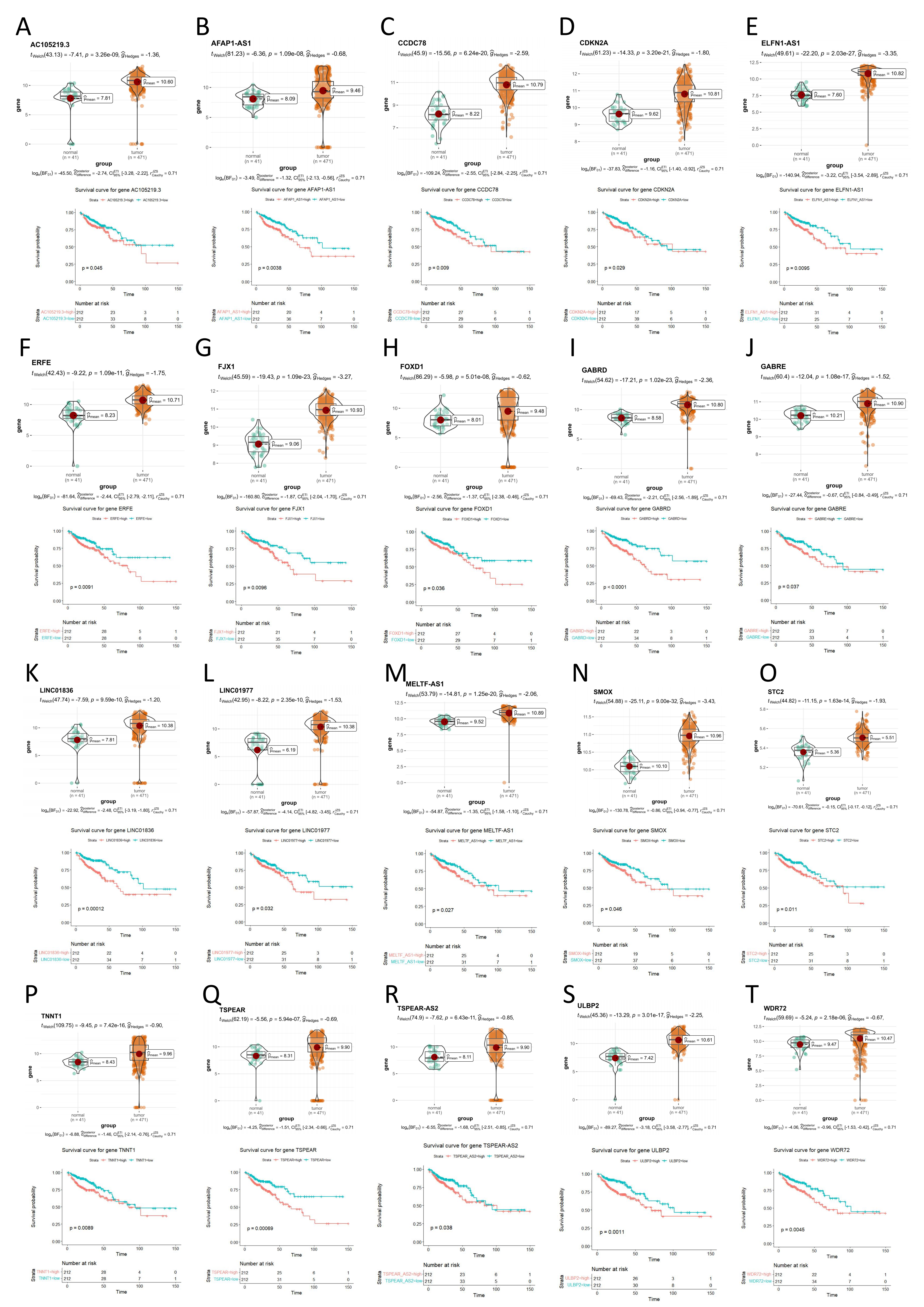

Supplement: Supplementary file 1 [file ijms-25-03954-s001.zip › Supplement Figure S1.tif]

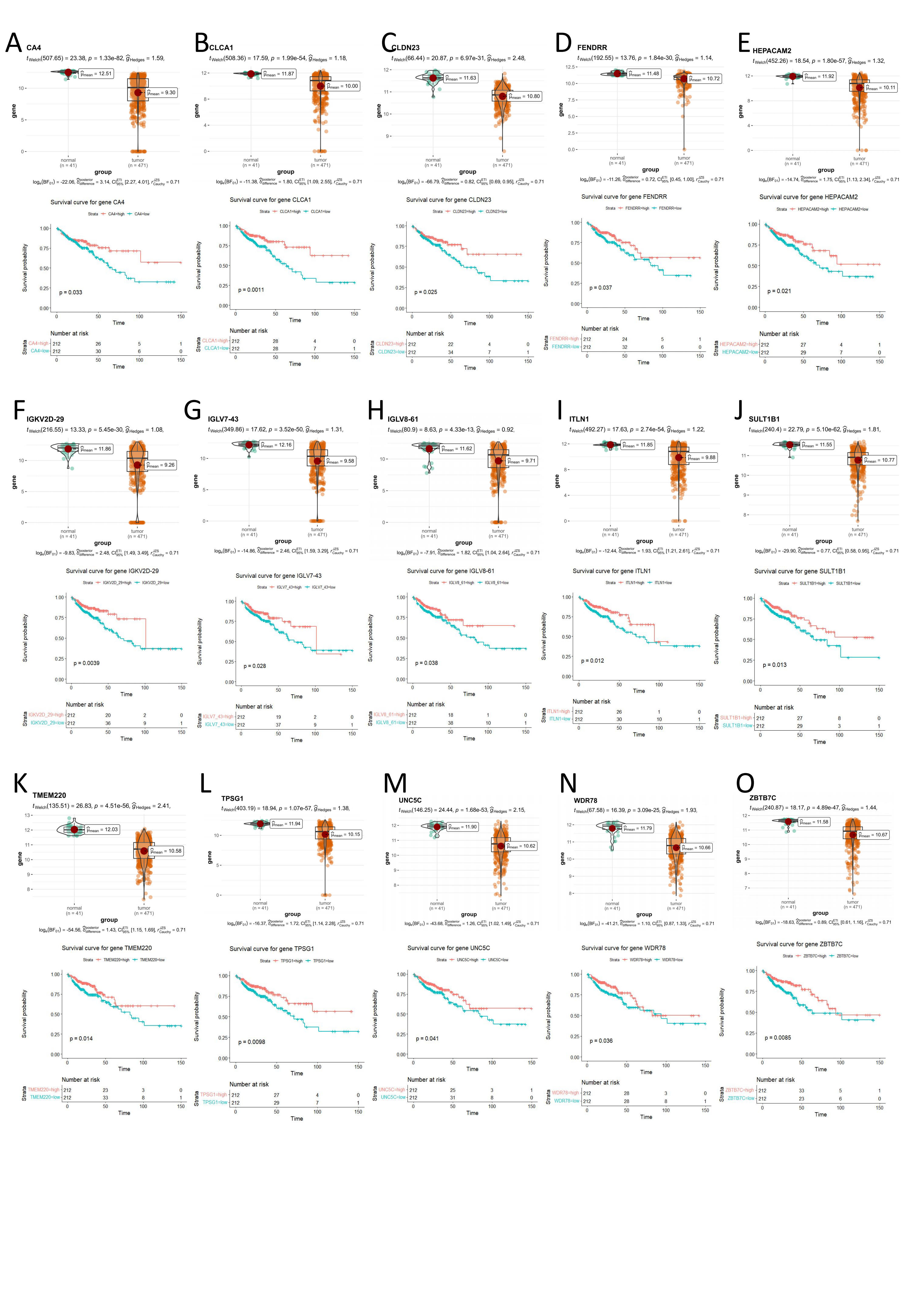

Supplement: Supplementary file 1 [file ijms-25-03954-s001.zip › Supplement Figure S2.tif]

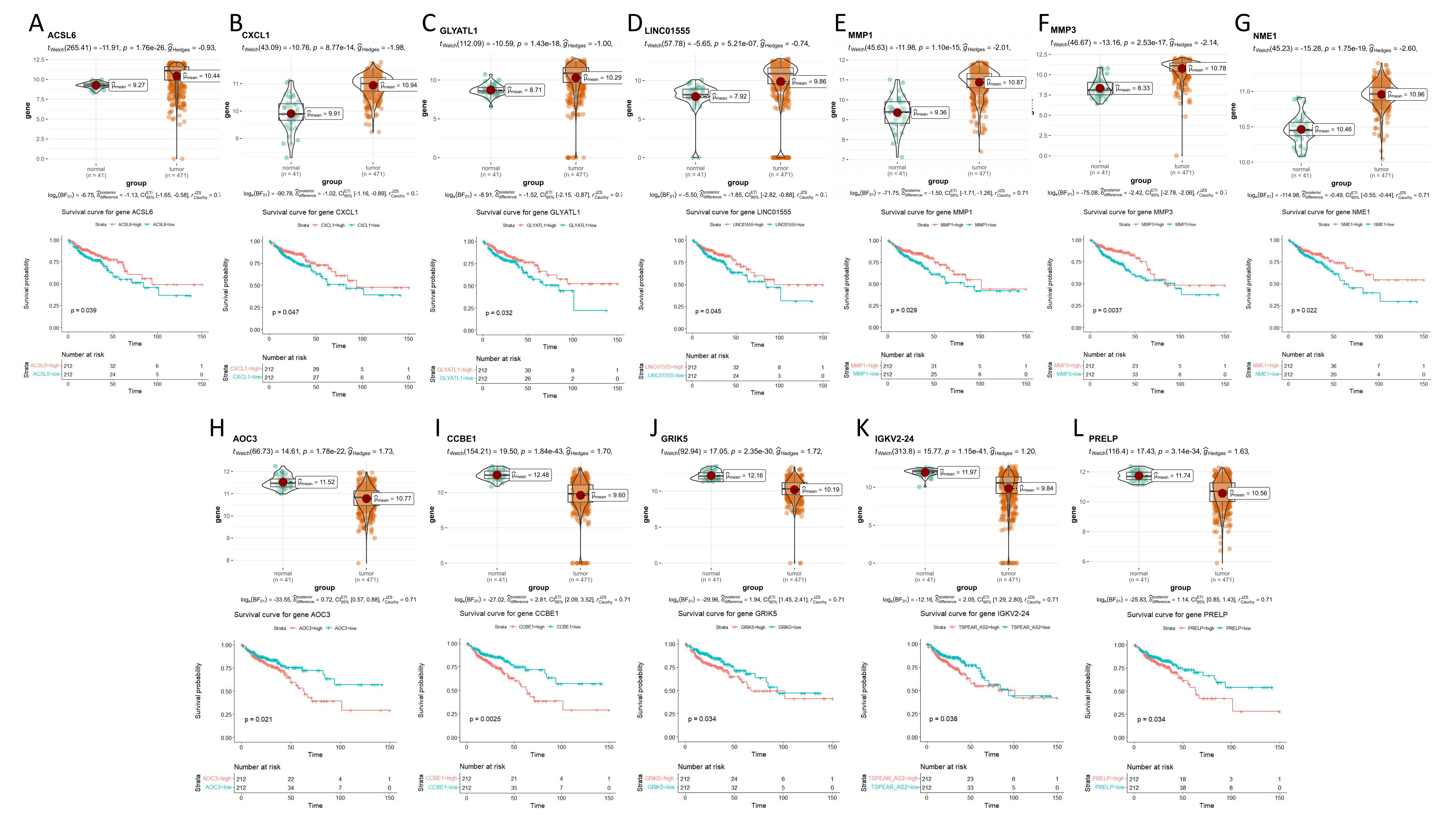

Supplement: Supplementary file 1 [file ijms-25-03954-s001.zip › Supplement Figure S3.tif]
